# Adaptation of Bacteria to Antineoplastic Agents Involves Persister Cells and Increases Resistance to Antibiotics

**DOI:** 10.3390/bioengineering9080355

**Published:** 2022-07-30

**Authors:** Carla C. C. R. de Carvalho

**Affiliations:** 1 iBB-Institute for Bioengineering and Biosciences, Department of Bioengineering, Instituto Superior Técnico, Universidade de Lisboa, Av. Rovisco Pais, 1049-001 Lisbon, Portugal; ccarvalho@tecnico.ulisboa.pt; Tel.: +351-21-841-9594; 2 Associate Laboratory i4HB—Institute for Health and Bioeconomy, Instituto Superior Técnico, Universidade de Lisboa, Av. Rovisco Pais, 1049-001 Lisbon, Portugal

**Keywords:** mitomycin C, cyclophosphamide, etoposide, methotrexate, cancer, tolerance, resistance, persistence, antineoplastic agents, antibiotics

## Abstract

The increasing number of life-threatening infections observed in cancer patients has been ascribed to chemotherapy-induced neutropenia and to invasive medical procedures such as surgery and the application of catheters. In this study, it was questioned if the infections could also be favored by an increased resistance of bacteria due to the adaptation to antineoplastic agents used in chemotherapy. After exposure to several antineoplastic agents, it was observed that cells of *Staphylococcus aureus*, *Mycobacterium vaccae*, *Pseudomonas aeruginosa,* and *Escherichia coli* changed the fatty acid profile of their cellular membranes, produced exopolymeric substances, and formed aggregates that adhered to surfaces. Additionally, when exposed to high concentrations of these compounds, a persister sub-population could be identified. After adaptation to antineoplastic agents, the minimum inhibitory concentration (MIC) of several antibiotics increased considerably in the tested strains.

## 1. Introduction

It has been estimated that 30% of patients receiving chemotherapy for nonhematologic neoplasms and 85% of patients administered induction chemotherapy for acute leukemias develop potentially life-threatening infections [1]. The risk for sepsis in cancer patients is, ca., ten times the risk observed for the general population [2], and infection is the second cause of death in cancer patients [3]. The high rate of infections in cancer patients has been ascribed to a lower ability of the host defense system, to the invasive procedures (e.g., surgery and application of catheters), and to the chemotherapy they have to endure [4,5]. Chemotherapy, radiotherapy, steroids, stem cell and bone marrow transplants, and some types of cancer can cause neutropenia, disrupt skin and mucosal barriers, and impair the reticuloendothelial function [6,7]. Infections can even be caused by micro-organisms that had been considered as nonpathogenic, by enteric bacteria such as *Escherichia coli* and *Enterobacter* species, or by bacteria usually found on the skin such as *Staphylococcus aureus* and *S. epidermidis* [8,9,10]. Both antibiotic and chemotherapy drugs have also been found to cause gut microbiota dysbiosis, which may increase the risk of resistant bacteria in the microbiota [3,11].

In 1973, Armstrong stated that altered host resistance, due to serologic or cellular factors, could allow relatively avirulent organisms, e.g., bowel flora, to cause lethal infections in cancer patients but he considered that the virulence of these microorganisms was unchanged when compared to the predisposition of those in cancer-free people [12]. Wang and co-workers suggested, in 2014, that a relation between intra-abdominal infections and the use of antibiotics against anaerobes could be a marker for precancerous lesions or early colorectal cancer [13].

In the late 1990s, a shift from Gram-negative to Gram-positive bacterial isolates was observed, with coagulase-negative staphylococci (CNS) and *S. aureus* causing most of the bloodstream infections in both leukemic and solid-tumor patients, and α-hemolytic streptococci and CNS causing the majority of nosocomial respiratory tract infections [14,15]. Cruz et al. also suggested recently that children should be tested for tuberculosis prior to chemotherapy, even in low-incident industrialized countries, to prevent fatalities related to tuberculosis in pediatric oncology patients and bone marrow transplant recipients [16].

The majority of the papers dealing with post-chemotherapy infections published so far concerned nosocomial infections [4,8,14,17], where it was implicit the presumption that the strains acquired by cancer patients were already adapted to antibiotics and/or biocidal agents. However, Meunier et al. showed that 10 of the 39 chemotherapeutic drugs they tested activated the SOS response in *Escherichia coli* [18]. The three drugs inducing the highest responses, dacarbazine, azacitidine, and streptozotocin, also contributed to the emergence of mutants of the commensals *S. aureus*, *Pseudomonas aeruginosa,* and *Enterobacter cloacae* resistant to first-line antibiotics. This study, thus suggested a complex interaction between cancer, chemotherapy, and gut microbiota. Another work by Hobson et al. analyzed the impact of anticancer chemotherapies on seven clinical isolates of KPC-type carbapenemase-producing *Enterobacteriaceae* [19]. It was found that certain chemotherapy drugs, such as azacitidine and dacarbazine, increased the frequency of resistant mutants and their MIC to ceftazidime-avibactam, used in the treatment of severe infections. Nevertheless, randomized controlled trails have also showed that antibiotic prophylaxis, started at the time of chemotherapy or neutropenia, reduces all-cause mortality, infection-related mortality, febrile episodes, and bacteraemias [20,21,22].

In recent years, the presence of persister cells within a population exposed to high concentrations of antibiotics has been acknowledged. These cells are able to survive when the majority of their counterparts are killed and were first described by Joseph W. Bigger in 1944 [23]. Cells in a “persister” state can survive prolonged drug treatment due to a slow-growth phenotype, present metabolic activity distinct from non-persister cells, and may constitute, ca. 1% of a bacterial population [24,25,26]. An important feature of persister cells is that they remain genetically susceptible to the reapplication of the antimicrobial drug: after stopping the drug treatment, the surviving persister population will re-establish a heterogeneous population (i.e., containing susceptible and persister cells) showing the same initial response to the antimicrobial drug [26,27,28]. The persister cells are, thus, genetically identical to the majority of the cell population but present a different tolerance phenotype. However, bacterial persistence may promote antibiotic resistance by increasing survival and mutation rates [29].

In this study, it was aimed to test the hypothesis that initially nonpathogenic antibiotic-susceptible bacteria are able to acquire tolerance/resistance mechanisms after exposure to antineoplastic agents administered to cancer patients. In particular, it was assessed if the tested bacteria could promote phenotypic adaptations related to the persister state, including changes in membrane composition and potential, and if these adaptations increased the tolerance to antibiotics. In this case, the patients could suffer from both nosocomial infections and from their own bacteria (e.g., *S. aureus*, *S. epidermis*, and *E. coli*) that could become antibiotic-tolerant/resistant after adaptation to the administered antineoplastic agents.

## 2. Materials and Methods

### 2.1. Bacterial Strains 

The Gram-positive strains *Staphylococcus aureus* ATCC 25923, *Mycobacterium aurum* CIP 104482, and *M. vaccae* ATCC 15483, and the Gram-negative bacteria *Pseudomonas aeruginosa* 8821M and *Escherichia coli* BL21(DE3)pLys were used in this study. 

### 2.2. Chemicals

The antineoplastic agents tested were the following: mitomycin C from *Streptomyces caespitosus* supplied by Sigma (Buchs, Switzerland); cyclophosphamide from Baxter Oncology GmbH (Halle, Germany); etoposide from PCH Pharmachemie (Haarlem, The Netherlands); methotrexate from Faulding Pharmaceuticals (Warwickshire, UK). The antibiotics used were the glycopeptides vancomycin and teicoplanin, the oxazolidinone linezolid, the synthetic fluoroquinolone levofloxacin, and the carboxyfluoroquinoline ciprofloxacin, all from Sigma-Aldrich (St. Louis, MO, USA).

### 2.3. Determination of MIC

The minimum inhibitory concentration (MIC) of each antineoplastic and antibiotic agent was determined by the broth microdilution method in 96-well microtiter plates according to the CLSI guidelines [30,31]. In short, the bacterial strains used for the inoculum were incubated overnight in Mueller–Hinton broth at 37 °C and 200 rpm. The exponentially growing cells were collected and diluted to a McFarland 0.5 turbidity standard. Serial dilutions of each compound to test were prepared using Mueller–Hinton broth in 96-well plates, so that the final volume was 150 μL per well. Aliquots of 50 μL of inoculum were added to each well. The plates were sealed with the Breath-Easy Sealing Membrane (Sigma-Aldrich, St. Louis, MO, USA) to prevent medium evaporation and incubated at 37 °C and 300 rpm in a Heidolph Inkubator 1000 (Heidolph Instruments, Schwabach, Germany). The optical density was read after 16 and 18 h in a Spectra Max 340PC plate reader from Molecular Devices (San Jose, CA, USA) at 600 nm. MIC was considered as the lowest concentration that inhibited bacterial growth.

To assess the resistance of cells to antibiotics adapted to antineoplastic agents, the cells were grown in the presence of 20 μL/mL of each antineoplastic agent, collected at the end of the exponential phase, washed with phosphate buffer (pH 7.0, 50 mM), and diluted to 0.5 McFarland, and the MICs of selected antibiotics were determined according to the CLSI guidelines, as described above.

Simultaneously, MICs were determined by spread-plating 10^4^–10^5^ cell cultures in agar plates containing the compound to be tested. Plates were incubated at the temperatures described previously and were observed after 18, 24, 36, and 48 h to assess colony development. 

All assays were performed in triplicate.

### 2.4. Tolerance to Antineoplastic Agent and Persister Formation

Exponentially growing cells were placed in contact with 500, 200, 100, 50, 25, 12.5, 6.3, 3.1, 1.6, and 0.8 μg/mL of each antineoplastic agent in 6-well microtiter plates containing Mueller–Hinton medium to assess the intrinsic tolerance of each strain to the antineoplastic agents tested. Samples were collected after 2, 4, and 24 h of exposure to the tested compounds. Assays, including blanks without the antineoplastic agents, were carried out in triplicate. The fatty acid composition of the cellular membranes was analyzed by gas chromatography. Cell viability and membrane potential were determined by fluorescence microscopy and image analysis, using fluorescent dyes. Culturable cells (cell forming units, CFUs) were determined by the spread plate technique. The size of cell clusters and biofilm formation were determined by microscopy and image analysis. The description of each technique is described in the following sections. The MIC of the adapted cells to each compound was determined as described in Section 2.3.

Persister formation was assessed by growing the cells at 37 °C and 300 rpm in 6-well microtiter plates covered with the Breath-Easy Sealing Membrane. The exponentially grown cells were challenged with 100 µg/mL of each antineoplastic and cell viability was accessed after 2 and 24 h of exposure by fluorescence microscopy and image analysis, as previously described [25]. Cellular growth of the persister cells was assessed both by measurement of the optical density (O.D.) at 600 nm and by CFU counts after plating on agar plates. 

All assays were performed in triplicate.

### 2.5. Biofilm Formation

The three-dimensional structure of the biofilms formed was assessed as described previously [32]. In summary, the biofilms formed in 6-well plates were observed under brightfield transmitted light and by using a strain-dependent calibration curve relating to the number of stacked cells and the intensity value in the image at a given position. The tridimensional structure of the biofilm was assessed by image reconstruction. The volume of each biofilm was calculated using the Surfer^®^ package from Golden Software (Golden, CO, USA). All assays were performed in triplicate and at least 15 images were acquired per data point.

### 2.6. Lipid Extraction and Determination of the Fatty Acid Composition

Bacterial cells were collected from the culture media by centrifugation and the lipids of the cells were extracted by a modified Bligh and Dyer method according to Findlay and colleagues [33]. The fatty acids were derivatized into fatty acid methyl esters and analyzed by gas chromatography on an Agilent (Santa Clara, CA, USA) 6890N gas chromatograph with an FID and a 7683 B series injector equipped with a 30 m HP-5 capillary column from J&W Scientific (Folsom, CA, USA), as described previously [34]. Peak identification was achieved by comparison of the retention times of the peaks and those of qualitative standards of bacterial fatty acid methyl esters and polyunsaturated fatty acid methyl esters (both from Supelco), and of a methyl *cis*-11-octadecenoate standard solution from Sigma-Aldrich. Peak identification was confirmed by injecting both standards and selected samples on a 5975B inlet MSD gas chromatograph–mass spectrometer from Agilent equipped with a DB-1 column from J&W Scientific. The results presented in this work are the average of three independent samples.

### 2.7. Cell Viability and Membrane Potential

Cell viability was studied using a LIVE/DEAD^®^ *Bac*Light^TM^ bacterial viability kit whilst the membrane potential was assessed using a *Bac*Light^TM^ bacterial membrane potential kit, both from Molecular Probes (Invitrogen Co., Carlsbad, CA, USA). The samples, prepared according to the respective protocol provided by the manufacturer, were observed by fluorescence microscopy using an Olympus CX40 microscope, with an Olympus U-RFL-T burner and a U-MWB mirror cube unit (excitation filter: BP450-480; barrier filter: BA515). Images were captured by an Evolution^TM^ MP5.1 CCD color camera using the software Image-Pro Plus, both from Media Cybernetics, Inc. (Rockville, MD, USA). Image analysis was carried out as described previously [35]. At least 15 images were taken from each sample.

### 2.8. Surface Tension of the Supernatant of the Cultures

Surface tensions of cell-free culture supernatants were measured using the ring tear-off method in a K8 tensiometer from Krüss GmbH (Hamburg, Germany), according to the Wilhelmy technique [36].

### 2.9. Error Analysis

The average error associated with the GC quantification of each FAME was ±2.1%, quoted for a confidence interval of 99.5%. The errors were calculated based on ten independently prepared standard solutions. The error associated with image analysis was ±3.8%, based on the standard deviation and sample mean of fifteen repeated images taken from the same sample, quoted for a confidence interval of 99.5%.

## 3. Results

### 3.1. Bacterial Tolerance to Antineoplastic Agents

Immunosuppressed cancer patients receiving antineoplastic agents, undergoing invasive procedures and with straight and indwelling catheters, are vulnerable to microbial infections. *E. coli* and *P. aeruginosa* are commonly isolated from leukemic and solid-tumor patients, and often present resistance to antibiotics [37,38,39]. However, Gram-positive bacteria have been found to be an important cause of infection in patients with cancer [14,40]. Among infections affecting cancer patients are those caused by rapidly growing mycobacteria [41] and *S. aureus* [40,42]. In the present study, strains of the Gram-negative species *P. aeruginosa* and *E. coli* and of the Gram-positive *M. aurum*, *M. vaccae*, and *S. aureus* were used.

To determine the tolerance of the tested strains to antineoplastic agents, MICs were determined after 18 h according to CLSI guidelines [30]. *S. aureus* cells were more susceptible to mitocycin C, a potent DNA crosslinker, than to any other antineoplastic agents tested, exhibiting an MIC of 3.25 μg/mL after 18 h of exposure (Table 1). Mitomycin C was also effective in killing both *P. aeruginosa* and *E. coli* cells, which could only endure a concentration of 12.5 μg/mL. The antineoplastic agents etoposide and methotrexate were the most efficient compounds in killing the *Mycobacterium* strains tested with MICs of 25 and 12.5 μg/mL, respectively (Table 1). All tested strains were quite tolerant to cyclophosphamide: for Gram-negative species, a MIC of 50 µg/mL was determined, whilst for Gram-positive species, a MIC of 100 µg/mL was observed.

### 3.2. Exposure to Antineoplastic Agents Induces Persistence

**Figure 1:**
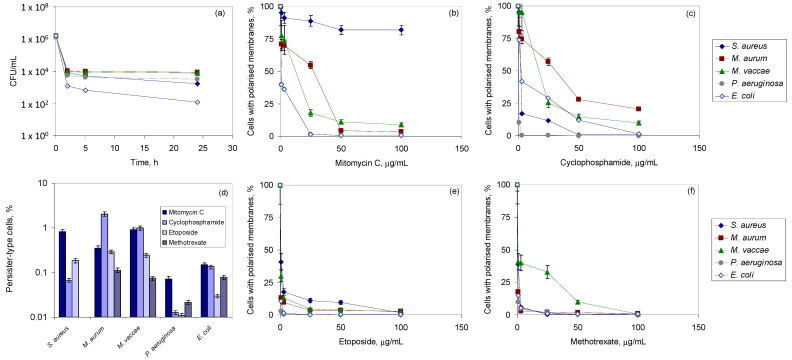
Persistence induced with antineoplastic agents. (**a**) Time-dependent killing curves during exposure to 100 μg/mL of mitomycin. Percentage of cells with polarized membranes following exposure to increasing concentrations of mitomycin (**b**), cyclophosphamide (**c**), etoposide (**e**), and methotrexate (**f**). (**d**) Percentage of persister-type cells observed after 24 h of exposure to 100 µg/mL of each antineoplastic for each tested strain. Results are the average of three independent bioconversions. Data are presented as mean ± standard deviation; assays were performed in triplicate.

The next step was to determine if the antineoplastic agents could induce the appearance of persister or resistant cells. For that, cultures in the mid-exponential phase were exposed to 100 µg/mL of each antineoplastic agent and the culture was monitored by fluorescence microscopy and CFU counts. Typical biphasic killing curves with a plateau or a slow decrease in CFUs were obtained in the present study, indicating the survival of the subpopulation of persister cells (example for cells exposed to mitomycin C in Figure 1a). Persister cells form a sub-population that is able to survive under conditions (e.g. in the presence of antibiotics) that kill the majority of their counterparts [23,27,43]. Contrarily to bacterial resistance, tolerance in persisters results from physiological processes rather than genetic mutations, the cells exhibiting slow growth rates, diminished membrane potential and protein synthesis, and changes in the lipid composition of their cellular membranes [25,28]. 

In the present study, a significant decrease in membrane potential was observed in all strains, except with *S. aureus* exposed to mitomycin C (Figure 1b,c,e,f). In this case, 81.8% of the cell population still presented a polarized membrane in the presence of 100 µg/mL of mitomycin C (Figure 1b), although a 2 log reduction in CFU was observed (Figure 1a). Etoposide and methotrexate affected greatly the proton motive force across the bacterial membranes of all strains (Figure 1e,f). Over 90% of the Gram-positive cells exposed to 3.1 µg/mL of these two antineoplastic agents presented depolarized membranes (Figure 1e,f). The exception was observed with *M. vaccae* cells that required 50 µg/mL of methotrexate to reach 90% of depolarized cells (Figure 1f). Both *P. aeruginosa* and *E. coli*, the Gram-negative strains tested, were highly affected by all antineoplastic agents, with at least 60% of the population presenting depolarized membranes for 3.1 µg/mL, but 0.8 µg/mL of etoposide and methotrexate hampered the proton motive force across the membrane of, ca., 90% of these cells (Figure 1e,f). 

The percentage of cells that survived exposure varied greatly with strain, and concentration and type of antineoplastic agent, but was, in general, less than 1% of the initial population (Figure 1d). As previously recommended [44], the cells that survived were placed in fresh media, exposed again to antineoplastic agents during the mid-exponential phase, and found to present the typical biphasic killing curves (data not shown). MICs were also determined and the same values as described in Table 1 were attained. This indicates that antineoplastics induced persistence but not resistance in the tested strains.

### 3.3. Antineoplastic Induced Changes in the Cellular Envelope

Modifications in the fatty acid (FA) composition of the phospholipids of the cellular membrane are a known adaptation mechanism for cell survival to toxic compounds and environmental conditions, as they allow cells to maintain the correct fluidity of their membrane [45,46]. These changes are also essential for the cells to enter a persister mode or a viable but not culturable state, and for the maintenance of their membrane potential [25,47]. 

**Figure 2:**
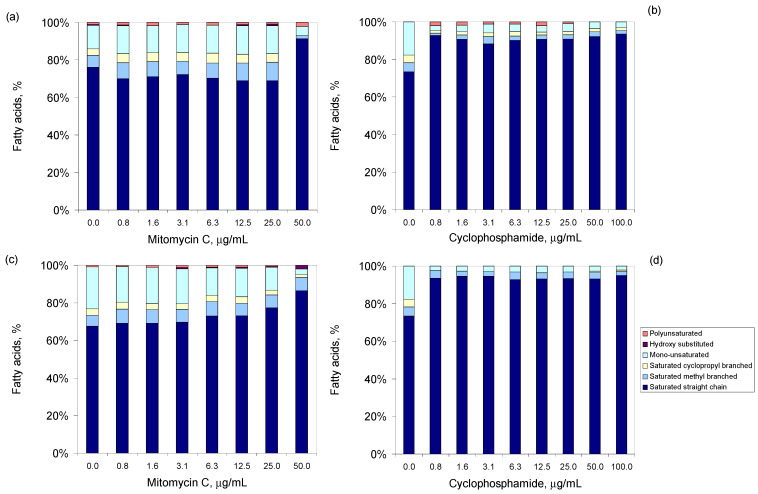
Fatty acid composition of the phospholipids of the cellular membrane of *S. aureus* cells exposed to mitomycin C (**a**,**c**) and cyclophosphamide (**b**,**d**), for 2 (**a**,**b**) and 24 h (**c**,**d**). Data presented are mean of three independent assays.

After an incubation period with the antineoplastic agents, changes in the FA composition of the phospholipids of the cellular membrane of the tested strains were observed (Figure 2). After an incubation of only 2 h, *S. aureus* exposed to up to 25 µg/mL of mitomycin C decreased the content of saturated straight FA by, on average, 7% when compared to unchallenged cells (Figure 2a). However, cells exposed to 50 µg/mL presented 21% more saturated straight FA. Additionally, the cells did not produce cyclopropyl-branched FA. This indicates that the cells responded to the toxicity of mitomycin C by decreasing the fluidity of their membrane. This response was clear when the *S. aureus* cells were exposed to cyclophosmide: a concentration of 0.8 µg/mL was sufficient for cells to increase the amount of saturated straight FA by 25% after a 2 h exposure, while decreasing 6.8-fold the amount of mono-unsaturated FA when compared to unchallenged cells (Figure 2b). After 24 h of exposure, a dose-dependent increase in the amount of saturated straight FA and a concomitant decrease in the amount of mono-unsaturated FA was observed for *S. aureus* cells exposed to both mitomycin C and cyclophosphamide (Figure 2c,d). The cells, thus, responded to the presence of these antineoplastic agents by increasing the degree of saturation of their membranes, resulting in a decrease in membrane fluidity that may hamper the entrance of the compounds into the cell. 

**Figure 3:**
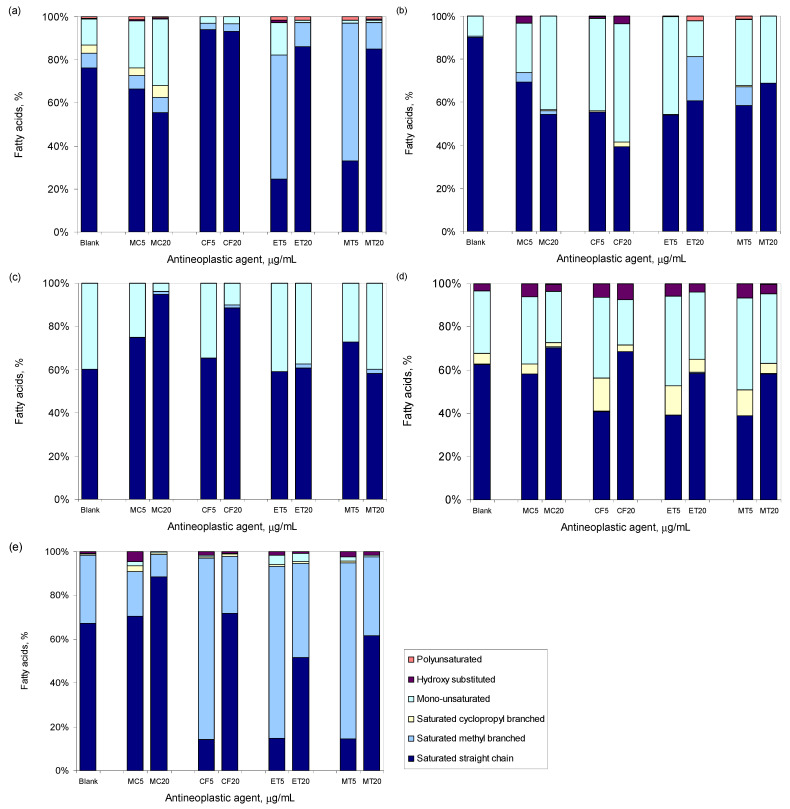
Fatty acid composition of *S. aureus* (**a**), *M. aurum* (**b**), *M. vaccae* (**c**), *E. coli* (**d**), and *P. aeruginosa* (**e**) following exposure for 24 h to 5 or 20 µg/mL of mitomycin C (MC), cyclophosphamide (CF), etoposide (ET), and methotrexate (MT). Blank refers to cells not exposed to antineoplastic agents. Data presented are mean of three independent assays.

Similar changes were observed for the other tested strains. To help the reader, only changes observed when the cells were exposed for 24 h to 5 and 20 µg/mL of each antineoplastic are shown in Figure 3. *M. aurum* responded to mitomycin by decreasing the amount of saturated straight-chain FA, similarly to *S. aureus* (Figure 3a,b). All other tested strains presented a dose-dependent increase in the content of these FAs while decreasing the content of mono-unsaturated FA (*M. vaccae* and *E. coli*) or saturated methyl branched FA (*P. aeruginosa*), when compared to unchallenged cells (Figure 3c–e). Diverse responses were observed when the cells were incubated with cyclophosphamide: opposite responses in membrane fluidity were observed between *M. aurum* and *M. vaccae*; both *P. aeruginosa* and *E. coli* presented membrane compositions closer to the unchallenged cells when exposed to 20 µg/mL than when exposed to 5 µg/mL (Figure 3a–e). As observed with *S. aureus*, etoposide and methotrexate induced the largest changes in membrane composition in comparison to control cells, and in general, the cells responded to these antineoplastic agents by decreasing the fluidity of the cellular membrane as a result of an increasing content of saturated straight FA. 

### 3.4. Antineoplastic Induced Other Adaptation Mechanisms

After an incubation of 24 h, all tested strains produced exopolymeric substances (EPS; data not shown). The substances produced had biosurfactant properties, decreasing the surface tension of the culture medium. For example, the EPS produced by *S. aureus*, *M. aurum*, and *E. coli* cells exposed to 0, 0.8, 6.25, and 25 µg/mL of mitomycin C for 24 h decreased the surface tension with increasing antineoplastic concentration, reaching 48.9, 57.3, and 49.8 mN/m for the highest concentration tested, respectively (data not shown). The initial broth presented a surface tension of 66.9 mN/m.

**Figure 4:**
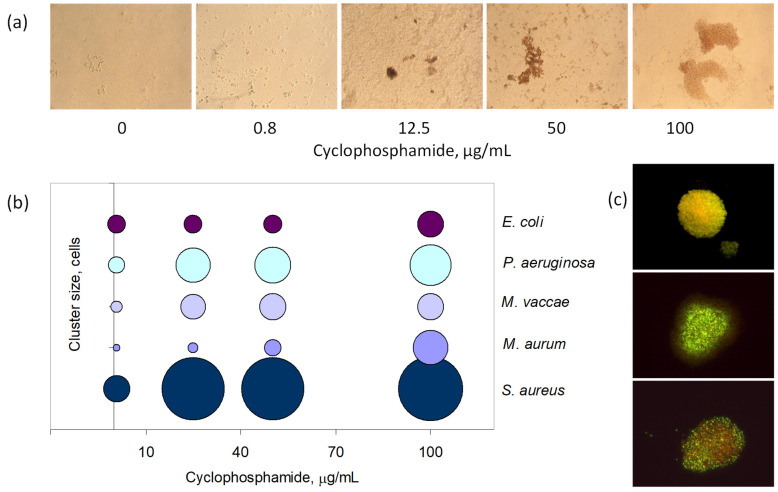
Cell clustering observed during exposure to antineoplastic agents. (**a**) Size of the *S. aureus* clusters observed during biofilm formation in 6-well plates in cultures with 0.8, 25, 50, and 100 μg/mL of cyclophosphamide, observed by brightfield microscopy (amplification: 150×). (**b**) Average cluster size observed in cultures of *S. aureus, M. aurum, M. vaccae, P. aeruginosa,* and *E. coli* exposed to 0.8, 25, 50, and 100 μg/mL of cyclophosphamide for 24 h. The diameter of the bubbles reflects the number of cells per cluster. (**c**) Clusters of *M. aurum* cells observed by fluorescence microscopy (amplification: 1500×). Healthy cells maintaining a membrane potential are stained red whilst those without a proton motive force across the membrane are stained green.

The production of surface-active compounds was accompanied by clustering of the cells (Figure 4c). This suggests that the cells were trying to protect themselves from the effect of the antineoplastic agents, as cells inside the clusters will be more protected than those on the outer layer of the aggregates [48]. Additionally, the average number of cells per cluster increased with the concentration of the tested compounds. In the case of cyclophosphamide, *S. aureus* cells exposed to 25 µg/mL formed clusters with 5.6-fold the number of cells of those exposed to 0.8 µg/mL (Figure 4b). *M. aurum* cells made clusters with 2.3 times more cells under the same conditions, and the clusters observed under 100 µg/mL of cyclophosphamide contained 20.9 times more cells than those observed with 0.8 µg/mL. *M. vaccae* and *P. aeruginosa* cells exposed to 100 µg/mL increased the number of cells per cluster, ca. 5-fold, whilst *E. coli* doubled the size of clusters when compared to cultures exposed to 0.8 µg/mL.

The production of EPS and the aggregation of cells resulted in the formation of biofilms, with the cells adhering to the surface of the multi-well plates (Figure 4a). The size of the adherent aggregates, during the first step of biofilm formation (adhesion of cells to the surface), reflected the concentration of the antineoplastic agent. The formation of biofilms has been related to the pathogenicity of some bacteria, such as staphylococci, and to the establishment of chronic infections due to bacterial protection from antibiotics and host defenses [49,50].

### 3.5. Cells Adapted to Antineoplastic Become Tolerant/Resistant to Antibiotics

To assess if the adaptation to antineoplastic agents could render the cells more tolerant to antibiotics, the bacterial cells were incubated with 20 µg/mL of each of the four antineoplastics tested, and the MIC of each antibiotic was determined and compared to that of cells not pre-exposed to antineoplastic agents. The antibiotics used were the glycopeptides vancomycin and teicoplanin, the oxazolidinone linezolid, the synthetic fluoroquinolone levofloxacin, and the carboxyfluoroquinoline ciprofloxacin. Vancomycin, teicoplanin, and linezolid are effective antibiotics against Gram-positive bacteria, levofloxacin is a broad-spectrum antibiotic with activity against both Gram-positive and Gram-negative bacteria, whilst ciprofloxacin is a potent antibiotic against Gram-negative bacteria but is also effective against several Gram-positive pathogens.

Cells grown in the presence of 20 μg/mL of mitomycin C presented significantly higher MICs to the tested antibiotics than nonexposed cells (Table 2). *S. aureus* cells presented a 16-fold increase in the MIC of ciprofloxacin, a 32-fold increase in the MIC of vancomycin, and a 42-fold increase in the MIC of levofloxacin, when compared to cells not pre-exposed to mitomycin. In the case of mycobacteria, *M. aurum* presented an 8-fold increase in the MIC for teicoplanin, whilst *M. vaccae* increased 42-fold the MIC for levofloxacin and 83-fold the MIC for ciprofloxacin. The Gram-negative *P. aeruginosa* showed an 8-fold increase in the MIC for ciprofloxacin, whilst *E. coli* increased the MIC for levofloxacin 4-fold. With the exception of the MIC for ciprofloxacin in *E. coli*., the adapted cells presented MIC values to the tested antibiotics that were at least double the values observed for cells nonadapted to antineoplastic agents. According to the MIC breakpoints provided by the European Committee in Antimicrobial Susceptibility Testing [51], the adapted bacteria tested were, in general, resistant to the antibiotics used. 

## 4. Discussion

Post-chemotherapy infections are responsible for a large number of fatalities among cancer patients, in addition to increasing the number of hospitalization days and costs [52,53]. These infections are usually considered to be nosocomial hospital-acquired infections resulting from neutropenia caused by chemotherapy agents [14,52,53]. Here, it was hypothesized that bacteria normally responsible for nosocomial infections could present higher resistance to antibiotics after exposure to antineoplastic agents.

Mitomycin C was effective in killing *S. aureus*, *P. aeruginosa,* and *E. coli* cells, which could only endure a concentration of 3.22–12.5 μg/mL (Table 1). A previous published study also demonstrated that treatment of *E. coli* B with 10 μg/mL of this antineoplastic agent completely blocks DNA formation in the cells [54]. It was shown that mitomycin C is able to kill persister cells by cross-linking DNA, in a process similar to that observed in actively growing cells [55]. In the present study, all tested strains were quite tolerant to cyclophosphamide (Table 1). This compound is probably the most frequently used immunosuppressive agent in experimental assays, and is a pro-drug requiring biotransformation to generate the active species.

Etoposide has been shown to inhibit DNA gyrase activity by trapping the gyrase-DNA complex at comparable levels in both *M. smegmatis* and *E. coli* [56], but the tested *E. coli* strain was more tolerant than expected. Similarly, a previous study reported that a strong antimicrobial activity of methotrexate, an inhibitor of dihydrofolate reductase, was only observed on *S. aureus* with a MIC90 of 20 and 100 μg/mL for the methicillin-sensitive and -resistant strains, respectively [57]. 

The results of the present study indicate that initially susceptible bacteria could adapt to the presence of the tested antineoplastic agents, with the population showing significant tolerance to both these compounds and to antibiotics (Table 1 and Table 2). The bacterial cells tested presented a significant intrinsic tolerance to the agents tested and, although the concentrations tested were sufficient to cause impairment of the proton motive force across the bacterial membranes, some cells could still maintain the membrane potential (Figure 1). The cells able to survive and thrive under conditions that kill the majority of the population are usually designated as persisters, which are not eliminated even by high doses of antibiotics [23,26]. They are one of the reasons why biofilms are difficult to eradicate and chronic infections flourish. The typical biphasic killing curves with a plateau of surviving cells (Figure 1) suggest that doses of biocides given according to the MICs determined at 18 or 24 h might not be sufficient to kill the slow growing persistent cells present in the population. 

Not only should persistent cells be a major concern to clinicians but also should regular, initially susceptible bacterial cells, since the present work shows that the latter were also able to adapt and grow following a 2 or 24 h exposure period to high concentrations of antineoplastic agents. The cells were able to produce extrapolymeric substances, to promote cell aggregation and the formation of biofilms (Figure 4), and to change the fatty acid composition of the cellular membrane to promote the suitable level of fluidity, in response to the presence of the antineoplastic agent tested (Figure 2). 

The bacterial membrane is usually the main target of organic compounds toxicity [58,59], with survival of the bacteria being dependent on its ability to maintain membrane fluidity and continued functionality by modulation of the fatty acid composition [46,60,61]. In bacteria, the degree of saturation, which is the ratio of saturated to unsaturated fatty acids, is the main mechanism bacteria possess to control membrane fluidity [62]. The presence of branched and cyclic fatty acids increases the fluidity of the membrane as their presence impedes the formation of crystalline structures among adjacent acyl chains [45,63].

The major response observed at the cellular membrane level of *S. aureus* cells to antineoplastic agents was an increase in the degree of saturation of the membrane and a decrease in the percentage of branched-chain fatty acids (Figure 3a). The increased percentage of saturated fatty acids and the lower amount of branched-chain fatty acids observed in exposed cells, in comparison to non-stressed cells, indicate that the cells were trying to decrease the membrane fluidity. In fact, branched-chain fatty acids and *anteiso* C15:0 (its concentration in the *S. aureus* membrane also decreased with increasing concentrations of mitomycin, cyclophosphamide, and methotrexate) are the major FA responsible for the fluidity of the membrane in *S. aureus* [64]. Changes in membrane structure and function, including enhanced membrane fluidity, increased net-positive surface charge, and translocation of positively charged phospholipids to the outer membrane leaflet, have been linked to in vivo development of daptomycin resistance in this bacterium [65,66].

The mycolic-acid containing *M. aurum* and *M. vaccae* cells responded to mitomycin, cyclophosphamide, and methotrexate differently: the former responded with alterations in the fatty acid profile with increasing concentrations of the antineoplastic agents to increase membrane fluidity, whilst *M. vaccae* adjusted the cellular membrane composition to decrease the fluidity of the membrane (Figure 3b,c). In fact, the adaptation abilities of these cells have allowed them to survive under harsh conditions and in the presence of quite toxic compounds [67,68]. 

In general, *E. coli* cells increased the percentage of saturated fatty acids with increasing concentrations of the antineoplastic compound, while decreasing the percentage of mono-unsaturated fatty acids (Figure 3d). The result was a less fluid membrane that should have acted as a better barrier against the entrance of toxic compounds in the cell. Nevertheless, the vast majority of the nonadapted *E. coli* population presented depolarized membranes after exposure to the tested compounds (Figure 1).

Considerable cell aggregation was observed with all tested strains after exposure to the antineoplastic agents, the clusters being, in general, larger with increasing concentrations (Figure 4). As the cells also started to produce exopolymeric substances after exposure, adherence of the cells and subsequent biofilm formation were observed (Figure 4). When the cells are embedded inside a matrix forming a biofilm, they are more protected against antimicrobials and antibiotics because of a slower growth rate and a lower diffusion rate of the compounds [69]. Cells in biofilms may even influence the leukocyte function of the host immune system [70] and the metabolism of immune cells, thus affecting the development and activation of immune reactions [71]. 

*S. aureus* is known to form biofilms, which is actually an important virulence mechanism allowing the cells to adhere to host tissues and to many medical devices, including catheters, causing acute and chronic infections. A previous study carried out in the USA estimated that around 32.4% of the population were colonized with *S. aureus* but the prevalence of methicillin-resistant strains in the isolates was estimated to be 0.84% for the same population [72]. However, a higher percentage of methicillin-resistant strains has been found to be nasally carried by healthcare workers [73,74]. 

Prophylactic treatment against febrile neutropenia and infection-related symptoms in patients with cancer has been found to be effective using levofloxacin [75,76]. In a systematic review of the infection-control interventions for cancer patients, it was found that protective isolation, including air quality control and barrier isolation, decreased the mortality of cancer patients but prophylactic antibiotics were found to be the most effective treatment [77]. However, the generalized use of prophylactic drugs in cancer patients should be well studied due to the associated costs, the application of unnecessary drugs to patients who might not develop neutropenia or infections, and the induction of more resistant bacteria. 

The results presented in this study clearly show that bacteria exposed to antineoplastic agents can respond to the challenge by using several phenotypic adaptation mechanisms, resulting in cells tolerant to both these agents and antibiotics (Table 1 and Table 2). The proliferation of naturally tolerant/resistant organisms can be seen in response to antibiotic treatment in both healthy and sick people [78], and in the present case, also in response to antineoplastic agents.

## 5. Conclusions

After exposure to compounds used in the treatment of patients with tumors and/or cancer, *S. aureus*, *M. vaccae*, *P. aeruginosa,* and *E. coli* changed the lipid profile of their cellular membranes, produced exopolymeric substances, and formed aggregates that adhered to surfaces (the initial step for biofilm formation). These adaptation mechanisms led to a several-fold increase in the MICs of the antibiotics vancomycin, teicoplanin, linezolid, levofloxacin, and ciprofloxacin. Vancomycin, teicoplanin, and linezolid are the last resort to treat, e.g., methicillin-resistant *S*. *aureus* infections, and the increase in MICs observed after adaptation to antineoplastic agents should be a major concern during oncology treatments. 

## Figures and Tables

**Table 1:** Minimum inhibitory concentrations of antineoplastic agents against the tested bacteria. MICs were determined in three independent assays.

	Minimum Inhibitory Concentration (MIC), μg/mL				
Compound	*S. aureus*	*M. aurum*	*M. vaccae*	*P. aeruginosa*	*E. coli*
Mitomycin C	3.25	100	100	12.5	12.5
Cyclophosphamide	100	100	100	50	50
Etoposide	100	25	25	100	100
Methotrexate	100	12.5	12.5	6.25	12.5

**Table 2:** Minimum inhibitory concentration of antibiotics against bacteria pre-exposed and not pre-exposed to the antineoplastic mitomycin C. MICs were determined in three independent assays.

MIC (µg/mL)	*S. aureus*	*M. aurum*	*M. vaccae*	*P. aeruginosa*	*E. coli*
Cells not pre-exposed to antineoplastic agents					
Vancomycin	5.2	2.6	5.2	-	-
Teicoplanin	2.6	10.4	2.6	-	-
Linezolid	2.6	5.2	2	-	-
Levofloxacin	2	41.7	2	5.2	10.3
Ciprofloxacin	2.6	41.7	2	20.8	41.7
Cells pre-exposed to antineoplastic agents					
Vancomycin	166.7	5.2	83.3	-	-
Teicoplanin	10.4	83.4	41.7	-	-
Linezolid	20.8	16.7	4.2	-	-
Levofloxacin	83.4	83.3	83.4	41.7	20.8
Ciprofloxacin	41.7	83.3	166.7	41.7	41.7

## Data Availability

The data presented in this study are available on request from the author.
